# Supplementary material for: Effect of Organic Anion Transporting Polypeptide 1B1 on Plasma Concentration Dynamics of Clozapine in Patients with Treatment-Resistant Schizophrenia
Source: Int J Mol Sci. 2024 Dec 9;25(23):13228. doi: 10.3390/ijms252313228 (PMC11642635; doi:10.3390/ijms252313228)
Supplement: Supplementary file 1 [file ijms-25-13228-s001.zip › ijms-3353945-supplementary.pdf]

Article

Effect of Organic Anion Transporting Polypeptide 1B1 on Plasma Concentration Dynamics of Clozapine in Patients with Treatment-Resistant Schizophrenia

Toshihiro Sato [1,*], Takeshi Kawabata [2], Masaki Kumondai [1,], Nagomi Hayashi [3], Hiroshi Komatsu [4], Yuki Kikuchi [5], Go Onoguchi [4,5], Yu Sato [1], Kei Nanatani [6,7], Masahiro Hiratsuka [1,3,6,7], Masamitsu Maekawa [1,3,6], Hiroaki Yamaguchi [8,9], Takaaki Abe [10,11,12], Hiroaki Tomita [4,5] and Nariyasu Mano [1,3]

1 Department of Pharmaceutical Sciences, Tohoku University Hospital, Sendai, Miyagi 980-8574, Japan; masaki.kumondai.d5@tohoku.ac.jp (M.K.); yu.sato.e7@tohoku.ac.jp (Y.S.); masahiro.hiratsuka.a8@tohoku.ac.jp (M.H.); m-maekawa@tohoku.ac.jp (M.M.); nariyasu.mano.c8@tohoku.ac.jp (N.M.)
2 Graduate School of Information Sciences, Tohoku University, Sendai, Miyagi 980-8578, Japan; takeshi.kawabata.b8@tohoku.ac.jp (T.K.)
3 Faculty of Pharmaceutical Sciences, Tohoku University, Sendai, Miyagi 980-8578, Japan; hayashi.nagomi.q7@dc.tohoku.ac.jp
4 Department of Psychiatry, Tohoku University Hospital, Sendai, Miyagi 980-8574, Japan; hkomatsu1019@gmail.com (H.K.); goh.ong.1008@gmail.com (G.O.); htomita@med.tohoku.ac.jp (H.T.)
5 Department of Psychiatry, Graduate School of Medicine, Tohoku University, Sendai, Miyagi 980-8575, Japan; ykikuchi@sand.ocn.ne.jp
6 Advanced Research Center for Innovations in Next-Generation Medicine, Tohoku University, Sendai, Miyagi 980-8573, Japan; kei.nanatani.a7@tohoku.ac.jp
7 Tohoku Medical Megabank Organization, Tohoku University, Sendai, Miyagi 980-8573, Japan
8 Department of Pharmacy, Yamagata University Hospital, Yamagata 990-9585, Japan; hiroaki.yamaguchi@med.id.yamagata-u.ac.jp
9 Graduate School of Medical Science, Yamagata University, Yamagata 990-9585, Japan
10 Division of Nephrology, Endocrinology, and Vascular Medicine, Graduate School of Medicine, Tohoku University, Sendai, Miyagi 980-8574, Japan; takaabe@med.tohoku.ac.jp
11 Division of Medical Science, Graduate School of Biomedical Engineering, Tohoku University, Sendai, Miyagi 980-8579, Japan
12 Department of Clinical Biology and Hormonal Regulation, Graduate School of Medicine, Tohoku University, Sendai, Miyagi 980-8575, Japan
* Correspondence: Toshihiro Sato, Ph.D toshihiro.sato@tohoku.ac.jp; Tel.: +81-22-717-7541; Fax: +81-22-717-7545

Citation: Sato, T.; Kawabata, T.; Kumondai, M.; Hayashi, N.; Komatsu, H.; Kikuchi, Y.; Onoguchi, G.; Sato, Y.; Nanatani, K.; Hiratsuka, M.; Maekawa, M.; Yamaguchi, H.; Abe, T.; Tomita, H.; Mano, N. Effect of Organic Anion Transporting-Polypeptide 1B1 on Plasma Concentration Dynamics of Clozapine in Patients with Treatment-Resistant Schizophrenia. Int. J. Mol. Sci. 2024, 25, x. https://doi.org/10.3390/xxxxx

Academic Editor: Giuliano Ciarimboli

Received: 20 November 2024
Revised: 4 December 2024
Accepted: 6 December 2024
Published: 9 December 2024

Copyright: © 2024 by the authors. Submitted for possible open access publication under the terms and conditions of the Creative Commons Attribution (CC BY) license (https://creativecommons.org/licenses/by/4.0/).

Supplementary Materials

Molecular Docking Calculations

The program AutoDock Vina 1.2.5 was used to perform molecular docking [61,62]. We followed the procedure for ensemble docking and prepared multiple conformations for both the ligand and the receptor. We performed molecular docking calculations for all possible combinations. For the receptor in the outward-open state, four experimental OATP1B1 structures were obtained from the Protein Data Bank (PDB): PDB_ID:8k6l, 8hnb, 8hnc, and 8hnh [34]. Whereas for the receptor in the inward-open state, two structures were obtained from PDB: PDB_ID:8hnd and 8phw [35]. Two conformations were prepared for the ligand molecule clozapine: the ideal conformation for PDB comp_id VBU and an experimental conformation taken from PDB (PDB_ID: 8jxv). Five conformations were prepared for the ligand molecule cyclosporin A: the ideal conformation for PDB BIRD PRD_000142, three experimental conformations from PDB (PDB_ID:1csa, 1ikf, and 2z6w), and one conformation from Cambridge Structural Database (CSD_ID: DEKSAN), based on previous simulation studies [63,64]. The PDB ideal conformation in mol2 format was flexibly aligned on the experimental structure in PDB format using the program *fkcombu* [65]. For each ligand–receptor conformation pair, the AutoDock program was run five times using different random number seeds. Each run generated 10 candidate binding poses of the ligand. The ligand poses were searched with exhaustiveness = 64, using flexible ligand conformations (except the ring structure) and the rigid receptor conformation.

The box for the binding site was determined by the pocket finding program *ghecom* using the largest cluster of pocket grids detected using R_{small}=1.87 Å and R_{large}=6 Å [66]. All the binding ligand poses generated from multiple ligand–receptor conformation pairs and multiple random seeds were clustered using the single linkage clustering method with the threshold RMSD of ligand atoms = 2.0 Å. The pose with the lowest "affinity" value was chosen for each cluster as the representative pose.

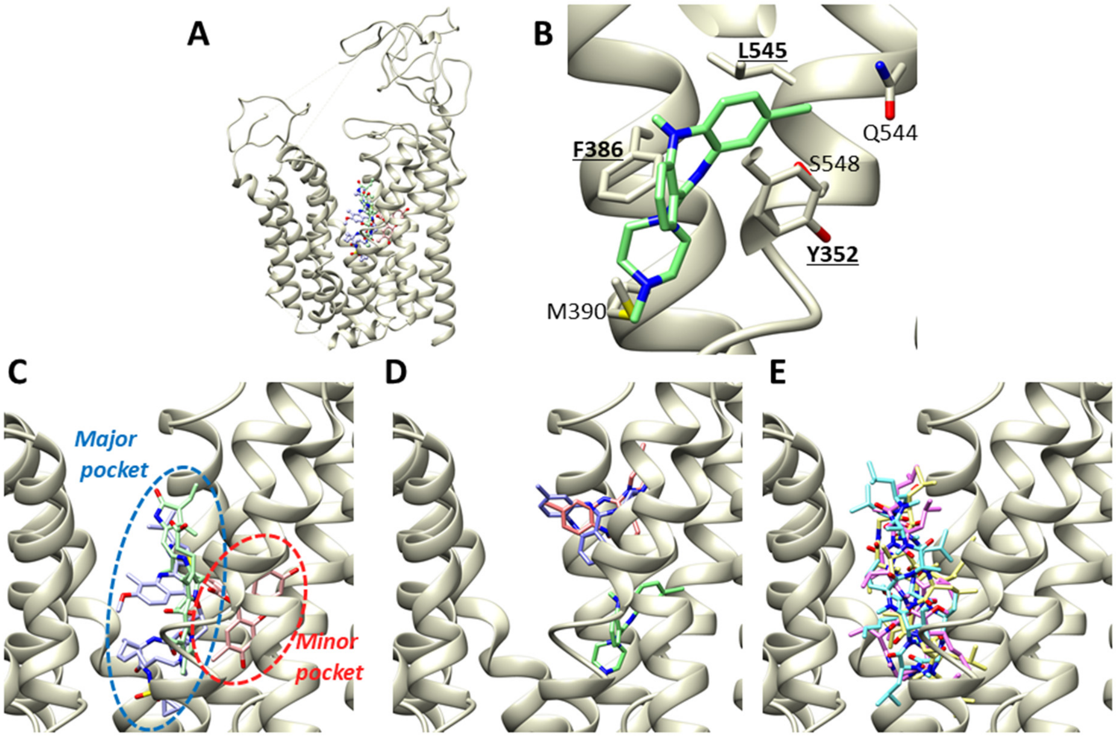

Figure S1. D structure of OATP1B1 in outward-open state with predicted binding poses of ligands. (**A**) Cryo-EM structure of OATP1B1 in outward-open state (PDB ID: 8k6l) with three ligands. (**B**) Predicted binding pose of CLZ on OATP1B1 in outward conformation. Representative conformation of the 3-rd rank cluster was shown. An amino acid of OATP1B1 in three predicted binding sites (Y352, A355, and L545) was different from those of OATP1B3 and OATP2B1 (Supplementary Figure 4). (**C**) Experimental structure of several binding molecules on OATP1B1 in outward-open state. 2',7'-dichlorofluorescein (comp_id: IOQ; PDB_ID: 8k6l) in "minor" pocket is shown in red; bilirubin IX alpha (comp_id: BLR; PDB_ID: 8hnc) and simeprevir (comp_id: 30B; PDB_ID: 8hnh) in "major" pocket are shown in green, and blue, respectively. (**D**) Predicted binding poses of CLZ. Generated poses were clustered and ranked by the best affinity value. The representative pose of the 1-st, 2-nd, and 3-rd clusters are shown in red, blue, and green, respectively. (**E**) Predicted binding poses of cyclosporin A. The representative pose of the 1-st, 2-nd, and 3-rd clusters are shown in yellow, cyan, and purple, respectively.

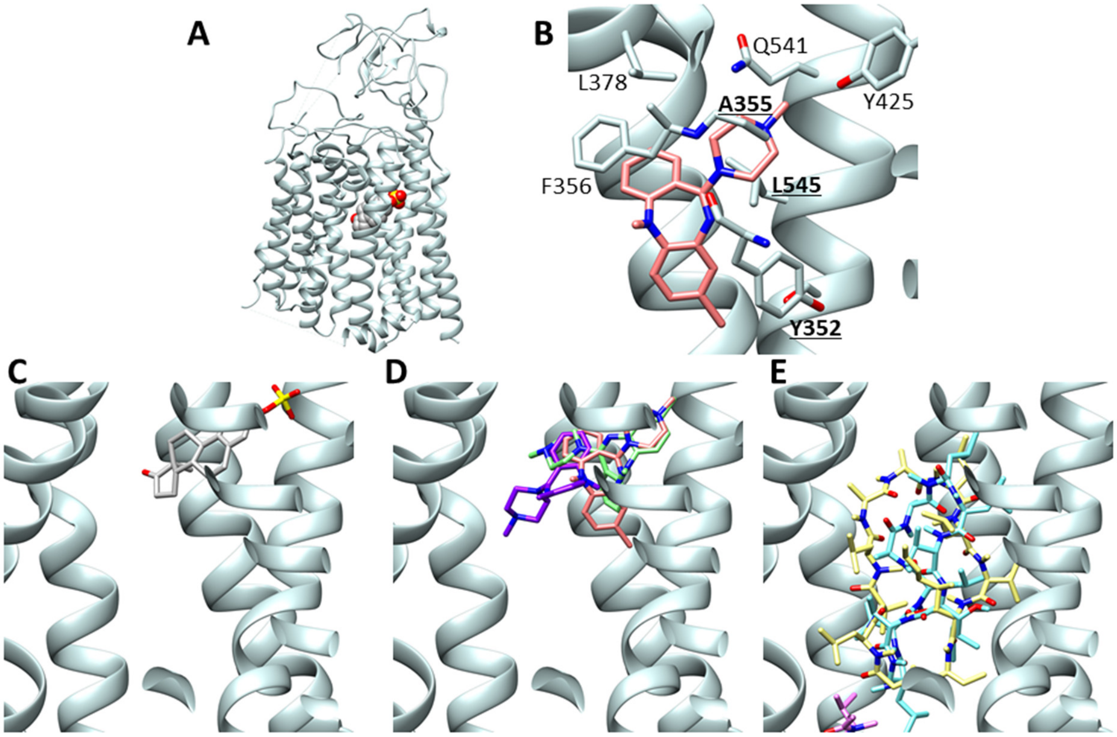

Figure S2. 3D structure of OATP1B1 in inward-open state with predicted binding poses of ligands. (A) Cryo-EM structure of OATP1B1 in inward-open state (PDB ID: 8hnd) with estrone 3-sulfate. (**B**) Predicted binding pose of CLZ on OATP1B1 in inward conformation. Representative conformation of the 1st rank cluster was shown. An amino acid of OATP1B1 in three predicted binding sites (Y352, F386, and L545) was different from those of OATP1B3 and OATP2B1 (Supplementary Figure 4). (**C**) Experimental structure of OATP1B1 in inward-open state with estrone 3-sulfate (comp_id: FY5; PDB_ID:8hnd). (**D**) Predicted CLZ binding poses. Generated poses were clustered and ranked based on the best affinity value. The representative pose of the 1-st, 2-nd, and 3-rd clusters are shown in red, blue, and green, respectively. (**E**) Predicted binding poses of cyclosporin A. The representative pose of the 1-st, 2-nd, and 3-rd clusters were shown in yellow, cyan, and purple, respectively.

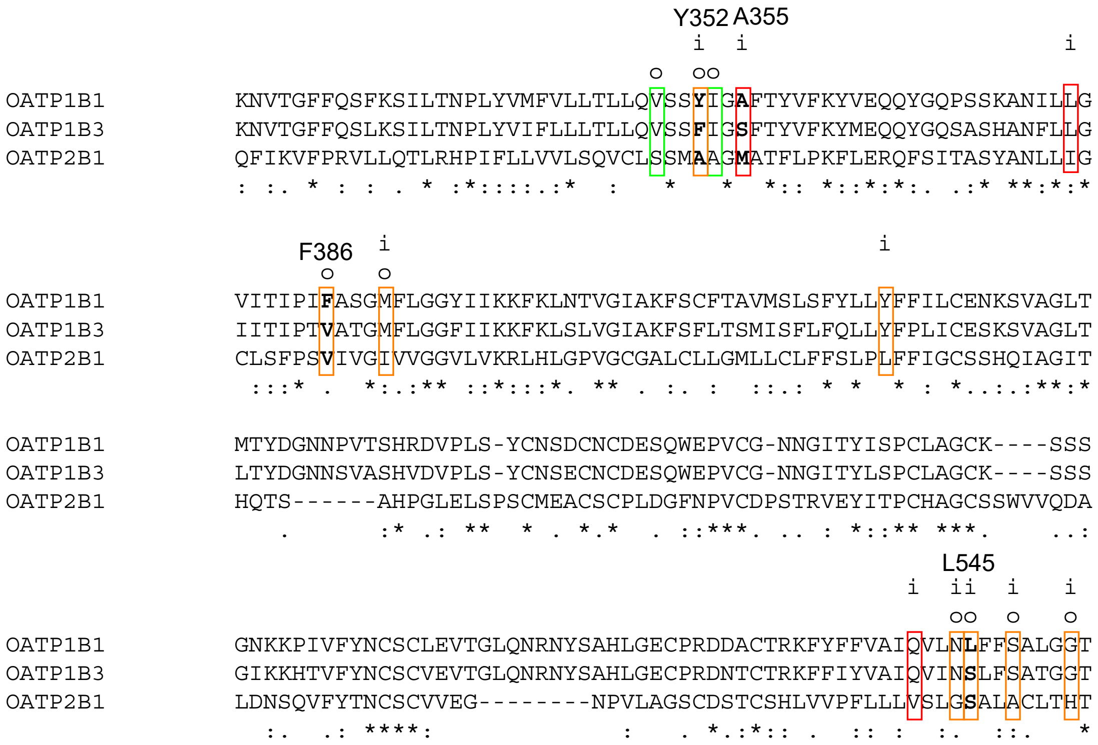

Figure S3. Multiple sequence alignment of OATP1B1, OATP1B3, and OATP2B1 with predicted binding sites of CLZ. The alignment was generated using the ClustalW2 program [36]. The sites denoted as ○ and i were the predicted binding sites of CLZ on OATP1B1 in the outward-open state (○, Supplementary Figure 1B) and inward-open state (i, Supplementary Figure 2B), respectively. Amino acid of OATP1B1 in four predicted binding sites (Y352, A355, F386, and L545) was different from those of OATP1B3 and OATP2B1.

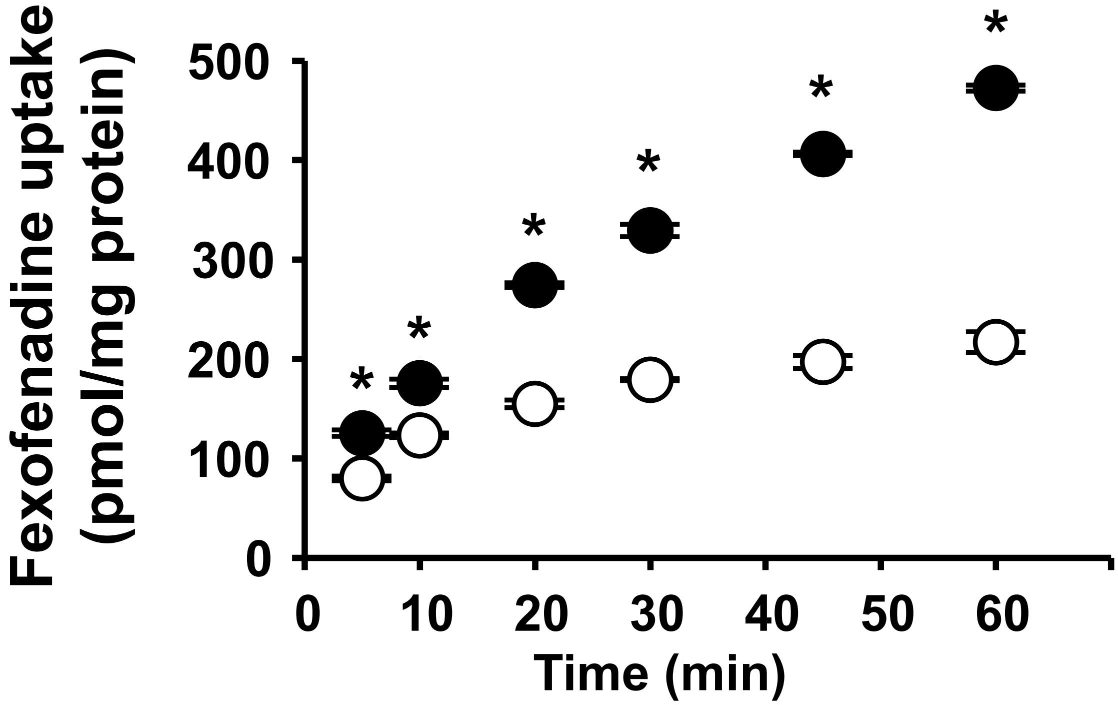

Figure S4. Time dependent fexofenadine uptake by newly established OATP2B1-expressing HEK293 cells. Cells were incubated with 1 μM fexofenadine for 5, 10, 20, 30, 45, and 60 min at 37 °C in KH buffer (pH 7.4). Open and closed circles represent mock cells (○) and OATP2B1-expressing HEK293 cells (●), respectively. An asterisk indicates a significant difference from the value of mock cells ($p < 0.05$). Data are presented as the mean ± standard error (S.E.) ($n = 3$).

Table S1 Raw data sets and calculation of plasma concentrations of CLZ, norCLZ, and other parameters of CLZ exposure and metabolism in 10 patients

Patient No.	Number of measurements	CLZ dose (mg)	Body weight (kg)	CLZ (ng/mL)	norCLZ (ng/mL)	nor-CLZ/CLZ	C/Dose (/L)	C/Dose/kg (/L•kg)
1	1	200	62.45	316	182	0.58	1.58	0.025
	2	200	61.8	282	197	0.70	1.41	0.023
	3	200	59.5	346	220	0.64	1.73	0.029
	4	200	59.15	248	158	0.64	1.24	0.021
2	1	300	44.65	717	262	0.37	2.39	0.054
	2	300	48.7	672	319	0.47	2.24	0.046
	3	300	47.55	395	138	0.35	1.32	0.028
3	1	175	31.2	347	174	0.50	1.98	0.064
4	1	400	58	683	551	0.81	1.71	0.029
5	1	500	46.2	616	342	0.56	1.23	0.027
	2	400	43.85	568	309	0.54	1.42	0.032
	3	425	43.05	536	324	0.60	1.26	0.029
	4	450	43.6	639	336	0.53	1.42	0.033
	5	450	43.1	472	242	0.51	1.05	0.024
6	1	200	75	378	303	0.80	1.89	0.025
	2	300	70.4	570	449	0.79	1.90	0.027
	3	400	65.05	336	335	1.00	0.84	0.013
7	1	200	56.7	803	434	0.54	4.02	0.071
8	1	325	NA	383.5	432.2	1.13	1.18	-
9	1	400	55	290.7	257.4	0.89	0.73	0.013
10	1	400	85	382.6	433.4	1.13	0.96	0.011
Average		320	55	475	305	0.67	1.59	0.031

NA, not available

Table S2 Primers list of drug metabolic enzymes analyzed in this study

Gene name	Position	Primer	Sequence (5'→3')
CYP1A2	Promoter ~ exon 2	Forward	GGAATCTTGAGGCTCCTTTCC
		Reverse	TCCAGGTCACACAGCTGGTC
CYP2C9	Exon 3	Forward	TGAAACCCATAGTGGTGCTG
		Reverse	CACAAATATGTGCAAATTCCCT
	Exon 7	Forward	GTGCATCTGTAACCATCCTCT
		Reverse	TAAGAGTAGCCAAACCAATCTT
CYP2C19	Promoter	Forward	GAGATCAGCTCTTCCTTCAGTTAC
		Reverse	AACCCAAGTGGTTCCAATGC
	Exon 2-3	Forward	GCTGAATATGTTGGTGTGAGG
		Reverse	CTCTCAGCTTCAAACCCTGC
	Exon 4	Forward	CTCAGTGCTTTGTTGTCTACAG
		Reverse	AAACAGGGCTTTGGAGTTTAGTGG
	Exon 5	Forward	AATAGGGCAGAGGTGTTTGATG
		Reverse	TCACAAATACGCAAGCAGTC
	Exon 7	Forward	GTGCATCTGTAGCAGTCCTCTC
		Reverse	CCCAGTGATGGTAGAGGGTAAG
CYP2A6	Whole	Forward	CCAGAAGGCTTTGCAGGCTTCA
		Reverse	ACTGAGCCCTGGGAGGTAGGTA
	Exon 1	Forward	GTGGGGGTGCCAGGTGTGTCCAGAGGAGCC
		Reverse	GGTAGGGGAGCCTCAGCACCTCTGCCGCCC
	Exon 2	Forward	AGTCTGGGGTGATCCTGGCTTGACAAGAGG
		Reverse	CACCCACCCGGGTCCCACGGAAATCTGTCT
	Exon 3-4	Forward	GTGGGGCTAATGCCTTCATGGCCACGCGCA
		Reverse	ACCTCTCGGGAGCTCGCCCTGCAGAGACTC
	Exon 5-6	Forward	GGTGAACGCAGAGCACAGGAGGGATTGAGA
		Reverse	CCTGTACCCTTCCTCCCTCGGCCCCTGCAC
	Exon 7-9	Forward	CAACATAGGAGGCAAGAAG
		Reverse	ATATAGCTCCCTGACGCC
CYP3A4	Exon 7	Forward	GGGTCTCTGTCTTCCTATGATG
		Reverse	GATGACAGGGTTTGTGACAGG
	Exon 10	Forward	GATGGCCCACATTCTCGAAG
		Reverse	AGATGAACCAGAGCCAGCAC
	Exon 11	Forward	GAGCACAGCAATGGGCATGAC
		Reverse	TCTCCATCTCTCCCTCTTTCTCC
CYP3A5	Intron 2	Forward	GTACCACCCAGCTTAACGAATGC

		Reverse	CCATTATGCCCAGCCTGTAG
FMO3	Exon 3	Forward	CTGTATCTGCCAAAACCATTTGCT
		Reverse	ATGTGCTATTTTAACACACCAAGC
	Exon 4	Forward	CTCACTTTTCACTCAGGACTAAACC
		Reverse	GGGGAAGCTCAGCTATGTGAAA
	Exon 5	Forward	GGGTGCTCACCAGAATATCCA
		Reverse	GGCATATCACGTTTAGCTCTGC
	Exon 6	Forward	GTCCTGAGGAAAGAGCCTGTAT
		Reverse	TACCCTTTATTACTTGTGCTGCCC

Table S3 Primers list of drug transporters analyzed in this study

Gene name	Position	Primer	Sequence (5'→3')
ABCB1	Exon 22	Forward	AGCATAGTAAGCAGTAGGGAGTA
		Reverse	TTGCAGGCTATAGGTTCCAGG
ABCG2	Exon 2	Forward	CCTGTGAGGTTCACTGTAGGT
		Reverse	CACCTAGTGTTTGCAATCTCATT
	Exon 5	Forward	ACCTTGGAGTCTGCCACTTT
		Reverse	AGCAGGCTTTGCAGACATCTAT
SLC22A8	Exon 7	Forward	CTATCACCCCTTCCTTGGCA
		Reverse	TCTCTTCGTCCTGCCCACTA
SLCO1B1	Exon 4	Forward	ACATCTCTTAAAACACATGCTGGG
		Reverse	ACGCGTAGTTTAAACCTGTGTT
	Exon 5	Forward	CACCATATTGTCAAAGTTTGCAAAGTGA
		Reverse	TTCAAAAGTAGACAAAGGGAAAGTGATCA
SLCO1B3	Exon 4	Forward	TTGGATCTAAACTACACAGACCGA
		Reverse	TCTCAAAAGGTAACTGCCCACT
	Exon 7	Forward	TGAAAACCAAGTATTTGTGACATCT
		Reverse	GGAAGAATGGTGTCCTGCACT
SLCO2B1	Exon 2	Forward	GGCCATTCTCGGGGATTCTA
		Reverse	TCTTCTAGACTCGGAGGCCC
	Exon 5	Forward	AGGTTTGTTGTGGGGCTCAA
		Reverse	GGGCTTCCCTGGACCTTTAC
	Exon 7	Forward	GGATGGCGGCTTAGAAGTGA
		Reverse	GAGAACAAGTGGGTGGTGCT
	Exon 10	Forward	ATCTCTGGCCATTGCTCTCG
		Reverse	TGGAGAGTATGACAGGCCCA
SLCO4C1	Exon 1	Forward	GTGAGAGCGCAAGCAAGTTC
		Reverse	CGCCCTCCCAAATCGAAGTC

References

34. Ciută, A.-D.; Nosol, K.; Kowal, J.; Mukherjee, S.; Ramírez, A.S.; Stieger, B.; Kossiakoff, A.A.; Locher, K.P. Structure of Human Drug Transporters OATP1B1 and OATP1B3. *Nat. Commun.* **2023**, *14*, 5774, doi:10.1038/s41467-023-41552-8.
35. Shan, Z.; Yang, X.; Liu, H.; Yuan, Y.; Xiao, Y.; Nan, J.; Zhang, W.; Song, W.; Wang, J.; Wei, F.; et al. Cryo-EM Structures of Human Organic Anion Transporting Polypeptide OATP1B1. *Cell Res.* **2023**, *33*, 940–951, doi:10.1038/s41422-023-00870-8.
36. Larkin, M.A.; Blackshields, G.; Brown, N.P.; Chenna, R.; McGettigan, P.A.; McWilliam, H.; Valentin, F.; Wallace, I.M.; Wilm, A.; Lopez, R.; et al. Clustal W and Clustal X Version 2.0. *Bioinformatics* **2007**, *23*, 2947–2948, doi:10.1093/bioinformatics/btm404.
61. Trott, O.; Olson, A.J. AutoDock Vina: Improving the Speed and Accuracy of Docking with a New Scoring Function, Efficient Optimization, and Multithreading. *J. Comput. Chem.* **2010**, *31*, 455–461, doi:10.1002/jcc.21334.
62. Eberhardt, J.; Santos-Martins, D.; Tillack, A.F.; Forli, S. AutoDock Vina 1.2.0: New Docking Methods, Expanded Force Field, and Python Bindings. *J. Chem. Inf. Model.* **2021**, *61*, 3891–3898, doi:10.1021/acs.jcim.1c00203.
63. Ono, S.; Naylor, M.R.; Townsend, C.E.; Okumura, C.; Okada, O.; Lee, H.-W.; Lokey, R.S. Cyclosporin A: Conformational Complexity and Chameleonicity. *J. Chem. Inf. Model.* **2021**, *61*, 5601–5613, doi:10.1021/acs.jcim.1c00771.
64. Yamane, T.; Ekimoto, T.; Ikeguchi, M. Development of the Force Field for Cyclosporine A. *Biophys. Physicobiology* **2022**, *19*, e190045, doi:10.2142/biophysico.bppb-v19.0045.
65. Kawabata, T.; Nakamura, H. 3D Flexible Alignment Using 2D Maximum Common Substructure: Dependence of Prediction Accuracy on Target-Reference Chemical Similarity. *J. Chem. Inf. Model.* **2014**, *54*, 1850–1863, doi:10.1021/ci500006d.
66. Kawabata, T. Detection of Multiscale Pockets on Protein Surfaces Using Mathematical Morphology. *Proteins Struct. Funct. Bioinforma.* **2010**, *78*, 1195–1211, doi:10.1002/prot.22639.
